# Impact of pre-transplantation exposure to immunosuppressive agents on lung transplant outcomes in interstitial lung disease

**DOI:** 10.3389/ti.2026.16549

**Published:** 2026-06-10

**Authors:** Karim Tazibet, Vincent Bunel, Hervé Mal, Tiphaine Goletto, Pierre Halitim, Lise Morer, Domitille Mouren, Mathilde Salpin, Adèle Sandot, Gaëlle Weisenburger, Philippe Montravers, Enora Atchade, Sébastien Tanaka, Justine Frija, Linda Hajouji, Yves Castier, Pierre Mordant, Bruno Crestani, Raphaël Borie, Kinan El Husseini

**Affiliations:** 1 Service de Pneumologie et Transplantation Pulmonaire, AP-HP, Hôpital Bichat, Paris, France; 2 Service de Pneumologie et Immuno-allergologie, Univ. Lille, CHU Lille, Lille, France; 3 INSERM, UMR 1149, Centre de Recherche sur l’inflammation, Université Paris Cité, Paris, France; 4 Service de Réanimation Chirurgicale, AP-HP, Hôpital Bichat, Paris, France; 5 Service de Physiologie, AP-HP, Hôpital Bichat, Paris, France; 6 Service de Réadaptation Respiratoire, AP-HP, Hôpital Bichat, Paris, France; 7 Service de Chirurgie Vasculaire et Thoracique, AP-HP, Hôpital Bichat, Paris, France

**Keywords:** corticosteroid therapy, immunosuppressants, interstitial lung disease, lung transplantation, selection criteria

## Abstract

Fibrosing interstitial lung diseases (ILDs) are the leading cause of lung transplantation (LTx), with worse results than other indications. We hypothesised that exposure to non-steroidal immunosuppressive agents (IAs) in the year preceding LTx for ILD may result in poorer early outcomes. We retrospectively analysed adults who underwent LTx for ILD from April 2011 to June 2024 in our institution and compared patients who received IA within 1 year before LTx to those who did not (systemic steroids exposure was used to adjust analysis). The primary outcome was 12-month retransplantation-free survival. Among 209 patients included, 76 (36%) had received IA within 1 year of LTx, and these patients had significantly worse 12-month retransplantation-free survival on multivariate analysis (62% vs. 80%; HR 1.99, 95%CI [1.11–3.56], p = 0.022); IA exposure increased the odds of grade 3 PGD (OR 3.20 [1.42–7.45], p = 0.005), bronchovascular fistula (OR 9.43, 95% CI [1.48–183], p = 0.042), pneumonia episodes in the first 6 months (median 2 [IQR 1-4] vs. 1 [0.5–2.5], p = 0.001), cytomegalovirus viremia under prophylaxis (21% vs. 5.2%, p = 0.005) and incidence of ganciclovir-resistant cytomegalovirus (14% vs. 3.2%, p = 0.021). In conclusion, IA exposure in the year before LTx leads to early complications and worse 12-month survival.

## Graphical Abstract

**Figure:**
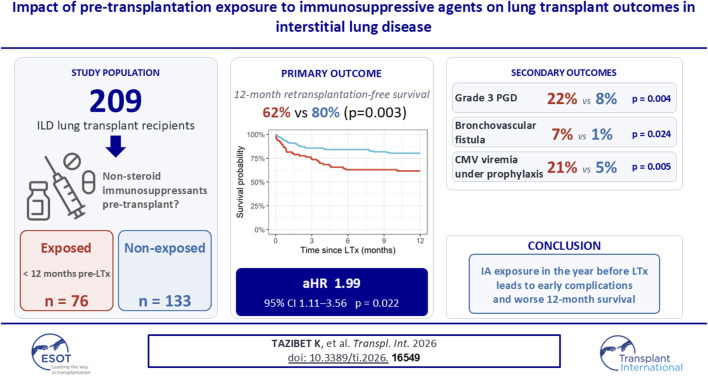

## Introduction

Fibrosing interstitial lung diseases (ILDs) are a heterogeneous group of respiratory diseases that can progress to terminal respiratory failure. Although they now represent the most frequent indication for lung transplantation (LTx) worldwide, they have the poorest prognosis, with a median survival of approximately 5 years after LTx [1]. Therefore, improving outcomes in this population is a critical unmet need.

The outcomes of LTx highly depend on the perioperative period, when severe complications can occur. Because this period can be affected by the preoperative characteristics of recipients, transplant teams strive to optimize candidate selection criteria. Treatment for patients with ILD before LTx may include corticosteroids, immunosuppressive agents (IAs, e.g., mycophenolate mofetil, rituximab, and cyclophosphamide), and antifibrotic agents [2, 3]. Retrospective studies have shown that corticosteroid treatment on the day of LTx is associated with increased infectious complications and poor survival [4], and many LTx centres now contraindicate LTx in patients receiving >20 mg prednisone equivalent per day [5]. The effect of antifibrotic therapies (pirfenidone, nintedanib) on LTx has also become a research focus [6, 7]. However, the impact of exposure to IA has not been specifically investigated. Given this lack of data, no specific guidelines have been established for IA use in the context of LTx [8].

Evidence suggests that the IA effects can persist beyond withdrawal, with varying intervals and severity depending on the treatment modalities [9, 10]. Hence, we hypothesized that prior treatment with IA may deepen the immunodeficiency of patients with ILD undergoing LTx, thereby increasing the risk of adverse outcomes after LTx, especially infectious complications.

This study aimed to determine whether early outcomes were worse for patients with ILD who underwent LTx after IA exposure in the year preceding LTx as compared with patients not exposed.

## Materials and methods

### Design

This was a single-centre retrospective study performed at the Lung Transplantation Centre of Bichat Hospital, Paris, France. We included all consecutive adults (>18 years old) who underwent LTx for chronic fibrosing ILD from April 2011 to June 2024. We excluded patients with sarcoidosis, interstitial diseases not classified as chronic fibrosing ILD (pulmonary alveolar proteinosis, hemosiderosis, acute respiratory disease syndrome), lung retransplantation for restrictive allograft syndrome (RAS), and multiple organ transplantations or if essential data were missing.

### Data collection and definitions

We relied on a local prospective database of all patients undergoing LTx and the electronic health records system at our institution. Data collected included patient demographics and pre- and post-LTx information.

Recent IA exposure was defined as IA treatment within the 12 months before LTx, as a dichotomous variable. IAs included biologic IAs (rituximab, tocilizumab, abatacept, anti-tumour necrosis factor) and non-biologic IAs (mycophenolate mofetil, azathioprine, cyclophosphamide, methotrexate, tofacitinib or tacrolimus, leflunomide, anti-JAK therapy). Systemic corticosteroids were not considered as IA but were recorded separately. The 12-month threshold was chosen to capture the clinically relevant peri-transplant immunosuppressive burden, encompassing agents with prolonged biological effects such as rituximab (B-cell depletion persisting months after the last infusion) while excluding remote exposures unlikely to affect peri-operative immunity.

Several variables were defined as follows: severe pulmonary hypertension (PH) as mean pulmonary artery pressure >20 mmHg and pulmonary vascular resistance (PVR) ≥5 Wood units (WU), or in the absence of PVR measurement, cardiac index <2 L/min/m^2^; pneumonia episode as a clinical suspicion of respiratory infection and evidence of new pulmonary infiltrates on chest imaging (chest X-ray or CT-scan), which was reviewed by the investigator; bronchovascular fistula as an abnormal connection between the bronchial lumen and adjacent thoracic blood vessels on bronchoscopy and CT-scan; acute cellular rejection (ACR) by a clinical suspicion supported by histological evidence or a favourable clinical and functional response to corticosteroid pulse therapy; allo-immunization as *de novo* donor-specific antibody detection with mean fluorescence intensity >1000; and acute antibody-mediated rejection (AMR), chronic lung allograft dysfunction (CLAD), bronchiolitis obliterans syndrome (BOS), RAS and primary graft dysfunction (PGD) as consensus reported [11, 12]. We defined grade 3 PGD as persistent PaO2/FiO2 ratio <200 or extracorporeal membrane oxygenation requirement (ECMO) for 72 h after LTx or death occurring earlier than 72 h with grade 3 PGD at the time of death. The LTx protocol at our institution is presented succinctly in Supplementary Methods, including bronchoscopy surveillance, maintenance immunosuppression and antimicrobial prophylaxis.

### Clinical outcomes

The primary outcome was 12-month all-cause mortality or retransplantation. Retransplantation was included in the composite primary endpoint as an event equivalent to death, representing definitive graft failure. Secondary outcomes were the occurrence of grade 3 PGD, occurrence of bronchovascular fistula, number of pneumonia episodes in the first 6 months, number of antibiotics courses in the first 6 months, invasive fungal infection in the first 6 months, occurrence of cytomegalovirus (CMV) viremia under prophylaxis and after prophylaxis withdrawal, occurrence of ganciclovir-resistant CMV, ACR, acute AMR within the first 12 months, forced expiratory volume in 1 s (FEV1) trajectory within the first 24 months, and all-cause mortality and CLAD-free survival at 36 months.

To evaluate infectious events during the first 6 months, only patients followed for at least 6 months were analysed. CMV donor-negative and recipient-negative (D-/R-) patients were excluded from CMV outcomes analysis. CMV viremia was defined as DNAemia over 3 log units/mL, which is the threshold for beginning curative treatment at our centre. FEV1 trajectory was analysed at each time point according to procedure laterality (single/double LTx).

### Statistical analysis

Variables are reported as median with interquartile range (IQR). We compared means for quantitative variables by unpaired Student’s t-test or Mann Whitney U test depending on the normality of the data distribution and chi-squared test or Fisher’s exact test for categorical variables (the normal distribution of variables was assessed with the Shapiro-Wilk test and by examining QQ plots). For multiple comparisons, we used ANOVA or the Kruskall-Wallis rank sum test. Survival analysis involved the log-rank test, Kaplan Meier curves and a Cox model for multivariate analysis. For multivariate analysis, covariables were selected according to clinical relevance, statistical significance (p < 0.1) and number of events to avoid overfitting. Preoperative patient characteristics were prioritized for survival analysis adjustment considering the focus of our study on pre-LTx risk factors and the expected collinearity between post-operative outcomes and early death. As a sensitivity analysis to assess the robustness of the primary finding to group imbalance, an inverse probability of treatment weighting (IPTW) analysis was performed. A propensity score for IA exposure was estimated using a logistic regression model including ILD subtype, severe pulmonary hypertension, acute exacerbation of ILD in the preceding 12 months, high emergency transplantation status, annual corticosteroid dose, age, and BMI. Stabilised average treatment effect (ATE) weights were derived and applied to a weighted Cox model with robust sandwich standard errors.

We conducted univariate logistic regression to assess the risk associated with early complications of LTx such as PGD and bronchovascular fistula. Competitive risk analysis was performed to analyse acute rejection risk and CMV viremia occurrence (with death as a competing risk), calculating cumulative incidence functions to describe the probability of events, with univariate regression analyses involving the Fine and Gray proportional-hazards model, estimating hazard ratios (HRs) and 95% confidence intervals (CIs). A multistate model was constructed, and transition probabilities were calculated for the analysis of CLAD-free survival at 36 months. A Wald-type Z-test was used to determine the statistical significance of differences in probability of events. Linear mixed models were fitted with FEV1 as a dependent variable; time (continuous), type of LTx (single/double) and optionally IA exposure as fixed effects; and individual as a random effect. Comparing these models allowed for assessing the effect of IA exposure on FEV1 trajectories. Correlation tests (Pearson or Spearman) were used to assess correlation between continuous variables. P < 0.05 was considered statistically significant. Statistical analyses were performed using the R environment R v4.2.0, The R Project for Statistical Computing.^1^

### Ethics

This observational study was designed, conducted, and written following the STROBE guidelines, and it received ethical approval from the French society of respiratory medicine (*Société de Pneumologie de Langue Française*) review board (CEPRO no. 2025-006).

### Patient and public involvement

Patients or members of the public were not involved in the design, conduct, reporting, or dissemination plans of this research.

## Results

### Population baseline characteristics

**FIGURE 1:**
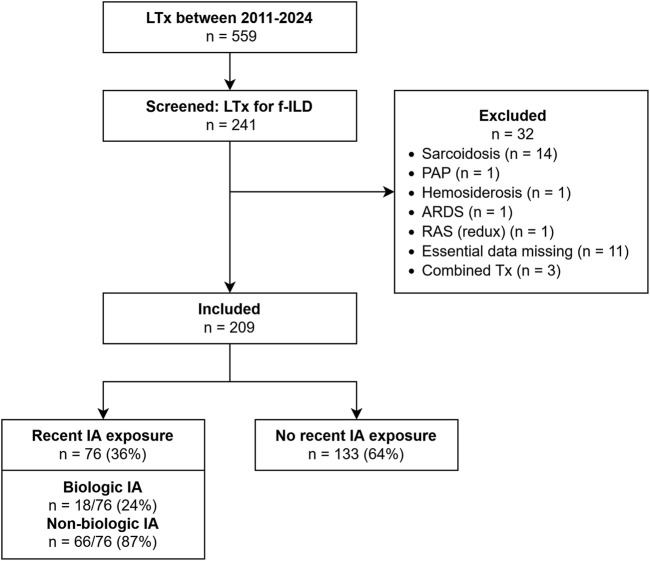
Flow chart of the study population. Essential missing data: related to immunosuppressive exposure and the time between their discontinuation and LTx. Biologic and non-biologic IA numbers do not add to 76 because more than one IA type was used in some patients. IA, immunosuppressive agent; f-ILD, fibrosing interstitial lung disease; PAP, pulmonary alveolar proteinosis; ARDS, acute respiratory distress syndrome; LTx, lung transplantation; RAS, restrictive allograft syndrome; recent IA exposure, last IA exposure <12 months before LTx; no recent IA exposure, no exposure or last IA exposure >12 months.

From April 2011 to June 2024, 559 LTx procedures were performed; 241 (43%) were for ILD. A total of 209 patients were included in this study; 76 (36%) had received IAs within 1 year of LTx (Figure 1). The most frequent IAs administered were cyclophosphamide (n = 25, 33%), mycophenolate mofetil (n = 33, 43%), azathioprine (n = 15, 20%), and rituximab (n = 15, 20%), with 17 (22%) patients having received two or more of these treatments during this interval (Supplementary Table S1). In the IA group, 49 (62%) patients were in the inter-cure phase, meaning they were within 6 months of their last dose of rituximab (n = 9, 12%), 28 days of cyclophosphamide (n = 4, 5%), and 2 weeks of tocilizumab (n = 2, 3%) and/or were receiving mycophenolate mofetil (n = 26, 34%), azathioprine (n = 10, 13%), tacrolimus (n = 2, 3%), tofacitinib (n = 2, 3%) or abatacept (n = 1, 1%) until the week of transplantation.

Pre-LTx baseline characteristics are summarized in Table 1. Patients with recent IA exposure were younger, more frequently women and had a higher body mass index. They more frequently had a diagnosis of connective tissue disease-ILD (CTD-ILD) or idiopathic non-specific interstitial pneumonia as opposed to idiopathic pulmonary fibrosis. They also had higher cumulative 12-month corticosteroid exposure (median 3.67 g [IQR 3.27–5.47] vs. 0 g [0–3.65], p < 0.001) and less frequently had received antifibrotics (28% vs. 62%, p < 0.001).

**TABLE 1:** Baseline and lung transplantation (LTx) characteristics by immunosuppressive agent (IA) exposure during the 12 months before LTx.

Characteristics	Total (N=209)	Recent IA (N=76)	No recent IA (N=133)	*p*-value
Age at transplantation (years)	58 (51–63)	56 (50–60)	59 (54–64)	**0.001**
Male sex, n (%)	159 (76)	50 (66)	109 (82)	**0.008**
BMI (kg/m^2^)	25.9 (23.4–28.7)	27.0 (23.5–29.7)	25.4 (23.4–28.1)	**0.04**
Ever-smoker, n (%)	139 (67)	46 (61)	93 (70)	0.2
Tobacco exposure (pack-years)	10 (0–25)	9 (0–25)	12 (0–25)	0.4
Comorbidities, n (%)				
Diabetes	28 (13)	12 (16)	16 (12)	0.4
Coronary disease	16 (7.7)	3 (3.9)	13 (9.8)	0.13
Arterial hypertension	42 (20)	14 (18)	28 (21)	0.6
GFR <60 (mL/min/m^2^)	7 (3.6)	5 (7.2)	2 (1.6)	0.1
ILD etiology, n (%)	​	​	​	**<0.001**
IPF	86 (41)	10 (13)	76 (57)	​
f-HP	31 (15)	12 (16)	19 (14)	​
CTD-ILD	37 (18)	31 (41)	6 (4.5)	​
f-NSIP	21 (10)	11 (14)	10 (7.5)	​
uILD	16 (7.7)	9 (12)	7 (5.3)	​
Other	18 (8.6)	3 (3.9)	15 (11)	​
Pre-LTx treatment				
Corticosteroids cumulative dose during the last 12 months (g)	3.00 (0.00–4.50)	3.67 (3.27–5.47)	0.00 (0.00–3.65)	**<0.001**
Corticosteroids treatment at transplantation	120 (57)	67 (88)	53 (40)	**<0.001**
Corticosteroids dosage at transplantation (mg)	5 (0–10)	10 (8–16)	0 (0–10)	**<0.001**
Any antifibrotics	103 (49)	21 (28)	82 (62)	**<0.001**
Antifibrotics treatment ongoing at transplantation	84 (40)	18 (24)	66 (50)	**<0.001**
Pre-transplantation lung function	​	​	​	​
Baseline FVC (%predicted)	44 (34–56)	41 (31–54)	46 (36–56)	0.1
Baseline DLCO (%predicted)	24 (18–32)	23 (15–28)	26 (20–33)	**0.009**
6-min walking test distance (m)	330 (240–435)	335 (230–420)	330 (240–450)	0.4
Right heart catheterization				
Pre-capillary PH	108 (52)	36 (48)	72 (55)	0.4
Severe PH	14 (6.9)	7 (9.6)	7 (5.3)	0.3
PAPm (mmHg)	23 (18–29)	23 (17–30)	24 (18–29)	>0.9
Type of lung transplantation	​	​	​	**0.014**
Double	114 (55)	50 (66)	64 (48)	​
Single	95 (45)	26 (34)	69 (52)	​
High emergency transplantation	64 (31%)	31 (41)	33 (25)	**0.016**
Acute exacerbation before LTx				
History of ILD exacerbation	72 (34)	31 (41)	41 (31)	0.14
Time since last exacerbation at transplant (days)	197 (34–343)	197 (27–338)	197 (40–359)	0.8
Donor age (years)	54 (42–64)	54 (43–64)	53 (41–63)	0.9
Preformed donor-specific antibodies	48 (23)	14 (19)	34 (27)	0.3
CMV mismatch	24 (12)	9 (12)	15 (12)	>0.9

Data are n (%) or median (interquartile range).

IA, immunosuppressive agents; BMI, body mass index; GFR, glomerular filtration rate; ILD, interstitial lung diseases; IPF, idiopathic pulmonary fibrosis; f-HP, fibrotic hypersensitivity pneumonia; CTD-ILD, connective tissue disease associated interstitial lung disease; f-NSIP, fibrotic non-specific interstitial pneumonia; uILD, unclassified interstitial lung disease; PH, pulmonary hypertension; MMF, mycophenolate mofetil; AZA, azathioprine; FVC, forced vital capacity; DLCO, diffusing capacity of the lung for carbon monoxide; WU, Wood unit.

* Other ILD etiology: pleuroparenchymal fibroelastosis, silicosis, desquamative interstitial pneumonia.

Bolded values represents p<0.05.

### LTx procedures

More than half of procedures were bilateral (55%) (Table 1). LTx was performed with the French high emergency allocation protocol for 64 (31%) patients. Patients with recent IA exposure more frequently had bilateral LTx (66% vs. 48%, p = 0.014), had high emergency transplantation (41% vs. 25%, p = 0.016) (Table 1) and received more packed red blood cells intraoperatively (median 3 packs [1–5] vs. 2 [0–3], p < 0.001) than those not exposed (Table 2).

**TABLE 2:** Immediate perioperative outcomes by immunosuppressive agent (IA) exposure.

Characteristics	Total (N=209)	Recent IA (N=76)	No recent IA (N=133)	*p*-value
Number of RBC pack transfusions	2.0 (0.0–4.0)	3.0 (1.0–5.0)	2.0 (0.0–3.0)	**<0.001**
ECMO				
Intraoperative ECMO	163 (78)	62 (82)	101 (76)	0.3
Preoperative VV-ECMO	12 (5.7)	6 (7.9)	6 (4.5)	0.4
Intraoperative VV-ECMO	8 (3.8)	5 (6.6)	3 (2.3)	0.14
Postoperative VV-ECMO	37 (18)	18 (24)	19 (14)	0.087
Preoperative VA-ECMO	6 (2.9)	4 (5.3)	2 (1.5)	0.2
Intraoperative VA-ECMO	160 (77)	59 (78)	101 (76)	0.8
Postoperative VA-ECMO	43 (21)	22 (29)	21 (16)	**0.024**
Time on mechanical ventilation (days)	5 (1–20)	9 (1–25)	4 (1–15)	0.059
Tracheotomy	55 (26)	26 (35)	29 (22)	**0.043**
PGD grade 3*	28 (13)	17 (22)	11 (8.3)	**0.004**
Airways complications	​	​	​	​
Bronchovascular fistula	6 (2.9)	5 (6.7)	1 (0.8)	**0.024**
Bronchial dilatation procedures	52 (25)	18 (24)	34 (26)	0.8
Acute kidney injury in ICU	63 (30)	31 (41)	32 (24)	**0.011**
Dialysis in ICU	36 (17)	18 (24)	18 (14)	0.062
Re-transplantation	4 (1.9)	2 (2.7)	2 (1.5)	>0.9
Re-transplantation <12 months	3 (0.1)	1 (1.4)	2 (1.5)	>0.9

Data are n (%) or median (interquartile range).

ECMO, extracorporeal membrane oxygenation; VV, venovenous; VA, venoarterial; PGD, primary graft dysfunction; ICU, intensive care unit; RBC, red blood cell.

* Defined as a persistent PaO2/FiO2 ratio <200 or ECMO, requirement within 48–72 h after LTx, or death occurring within the first 48 h with grade 3 PGD. Bolded values represents p<0.05.

### Survival analysis

Overall median survival was 43 months (95%CI [33.7–64.4]), and 12-month retransplantation-free survival was 74% (Supplementary Table S2). Four patients (1.9%) underwent re-transplantation, 3 (1.4%) within the first 12 months after LTx (1 in the IA group).

**FIGURE 2:**
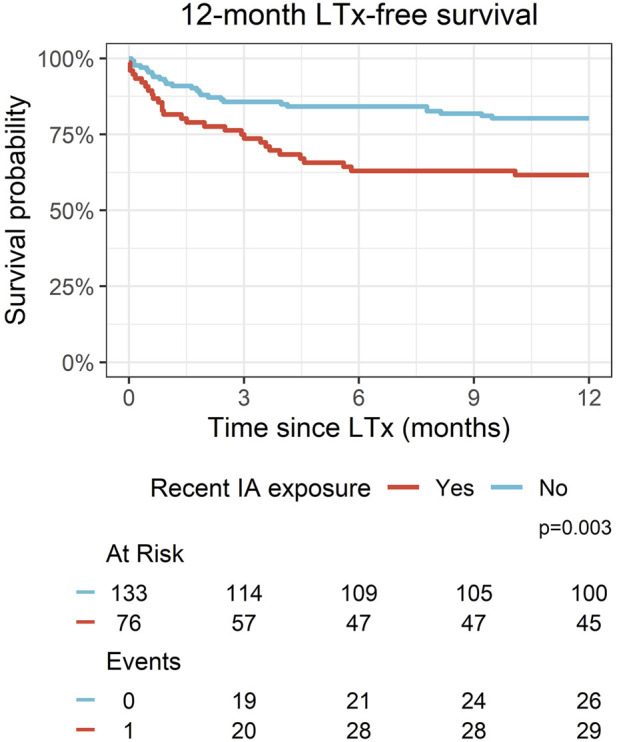
Twelve-month lung transplantation (LTx)-free survival by immunosuppressive agent (IA) exposure status (Kaplan-Meier curves). Recent exposure: <12 months before LTx.

Twelve-month re-transplantation-free survival was reduced with recent IA exposure on univariate analysis (62% vs. 80%, HR 2.21, 95% CI [1.30–3.76], p = 0.003) (Figure 2) and multivariate analysis (HR 1.99 [1.11–3.56], p = 0.022) after adjusting for severe PH, cumulative dose of corticosteroids, high emergency status and recent ILD acute exacerbation (Table 3). Preoperative ECMO was not included in the multivariable model, as it is collinear with high emergency status. In the IPTW sensitivity analysis (n = 204 with complete covariate data; 10 of 12 covariates achieved standardised mean differences <0.10 after weighting; Supplementary Table S3), the weighted HR of IA exposure for 12-month retransplantation-free survival was 2.44 (95% CI 1.27–4.70, p = 0.007), consistent with the primary multivariable estimate.

**TABLE 3:** Univariate and multivariate analysis of 12-month re-transplantation-free survival.

​	​	Univariate analysis			Multivariate analysis		
Characteristics	N	HR	95% CI	p-value	HR	95% CI	p-value
IA exposure <12 months	209	2.21	1.30–3.76	**0.003**	1.99	1.11–3.56	**0.022**
Corticosteroids cumulative dose (last 12 months) (g)	200	1.08	1.01–1.17	**0.029**	1.02	0.92–1.13	0.7
Corticosteroids at transplantation	209	1.33	0.77–2.31	0.3	​	​	​
Corticosteroids dosage at transplantation (mg)	209	1.01	1.00–1.02	0.14	​	​	​
Antifibrotics at transplantation	209	0.85	0.49–1.47	0.6	​	​	​
Male sex	209	1.77	0.87–3.62	0.12	​	​	​
Age at transplantation (years)	209	1.0	0.97–1.02	0.7	​	​	​
BMI ≥30 (kg/m^2^)	209	1.28	0.64–2.54	0.5	​	​	​
Severe pulmonary hypertension	204	2.30	1.04–5.10	**0.040**	1.77	0.77–4.07	0.2
ILD etiology	204	​	​	​	​	​	​
IPF	​	—	—	​	​	​	​
CTD-ILD	​	1.40	0.64–3.06	0.4	​	​	​
f-HP	​	1.34	0.58–3.10	0.5	​	​	​
f-NSIP	​	2.11	0.91–4.89	0.081	​	​	​
Other	​	1.14	0.38–3.39	0.8	​	​	​
uILD	​	3.45	1.49–8.01	**0.004**	​	​	​
ILD exacerbation <12 months	209	1.87	1.10–3.17	**0.021**	1.56	0.85–2.87	0.2
Type of LTx	209	​	​	​	​	​	​
Single	​	​	—	—	​	​	​
Double	​	​	1.09	0.64–1.86	0.8	​	​
High emergency transplantation	209	1.68	0.98–2.88	0.061	1.16	0.62–2.17	0.6

IA, immunosuppressive agent; CI, confidence interval; HR, hazard ratio; ILD, interstitial lung diseases; IPF, idiopathic pulmonary fibrosis; f-HP: fibrotic hypersensitivity pneumonia; CTD-ILD, connective tissue disease-associated interstitial lung disease; f-NSIP, fibrotic non-specific interstitial pneumonia; uILD, unclassified interstitial lung disease; LTx, lung transplantation. Bolded values represents p<0.05.

Notably, patients exposed to a non-biologic IA alone (n = 56) versus a biologic IA alone (n = 10) in the year before LTx did not differ in 12-month re-transplantation-free survival on multivariate analysis (n = 58) (HR 1.53 [0.57–4.14], p = 0.398) (Supplementary Table S4; Supplementary Figure S1). Considering the span of our study included the recent start of a systematic immunological induction protocol at our centre, we performed a sensitivity analysis excluding the patients who had received induction (Basiliximab n = 8, anti-thymocyte globulin n = 2), and found a similar impact of IA exposure on survival (HR 2.29 [1.22–4.30], multivariate p = 0.010). To account for other potential changes in practice over the years, we performed univariate Cox regressions stratified on LTx era. The prognostic effect of recent IA exposure was preserved in both early (2011-2018, n = 97, HR 2.38 [1.08–5.22], p = 0.031) and recent eras (2019-2024, n = 122, HR 2.08 [1.01–4.26], p = 0.046).

Finally, longer-term analysis using multi-state modelling incorporating CLAD risk demonstrated a higher probability of death at 36 months for patients with recent IA exposure before LTx (48% vs. 35.5%, p = 0.034) (Supplementary Table S5; Supplementary Figure S2).

### Immediate perioperative outcomes, PGD

Patiencs with recent IA exposure more frequently required postoperative veno-arterial ECMO (VA-ECMO) after surgery (29% vs. 16%, p = 0.024) and had grade 3 PGD (22% vs. 8.3%, p = 0.004) and acute kidney injury (41% vs. 24%, p = 0.011) (Table 2). Univariate logistic regression analysis revealed the following risk factors for grade 3 PGD: recent IA exposure (OR 3.20, 95% CI [1.42–7.45], p = 0.005), perioperative blood transfusion volume (OR 1.19, 95% CI [1.07–1.35], p = 0.003), and double LTx (OR 4.55 [1.78–1.04], p = 0.003) (Supplementary Table S6).

### Airway complications

Bronchovascular fistula was diagnosed in 6 patients (2.9%), mainly in the IA group (5/76 (6.7%) versus 1/133 (0.8%), p = 0.024) and recent IA exposure was the only factor associated with this severe complication in univariate analysis (OR 9.43, 95% CI [1.48–183], univariate p = 0.042) (Supplementary Table S7). Conversely, there were similar rates of bronchial stenosis requiring dilatation during the 12 months after LTx in both groups (Table 2).

### Early infectious outcomes

We included 156 patients with follow-up data for at least 6 months in the analysis. IA exposure was associated with increased number of pneumonia infections (median 2 [1–4] vs. 1 [0.5–2.5], p = 0.001) and antibiotics courses (4 [3–5] vs. 3 [1–5], p = 0.010) within 6 months post-LTx (Supplementary Table S8). The groups did not differ in *Clostridium difficile*, *Pseudomonas aeruginosa* or invasive fungal infections.

### CMV complications

Overall, 20 of 150 recipient-positive (R+) and donor-positive recipient-negative (D + R-) patients (13% of those at risk) experienced CMV viremia under prophylaxis and 60 (40%) after prophylaxis withdrawal. In a survival analysis with death as a competing risk, the cumulative incidence of CMV viremia under prophylaxis during the first 12 months after LTx was higher for patients with IA exposure (21% vs. 5.2%, p = 0.005) (Supplementary Figure S3; Supplementary Table S9). Also, ganciclovir-resistant CMV was more frequent in patients with recent IA exposure (14% vs. 3.2%, p = 0.021) (Supplementary Table S10).

### Immunological outcomes and allograft dysfunction

Preformed DSA was present in 14 patients (18.7%) in the IA group versus 34 (26.8%) in the non-IA group (p = 0.3), while 35 (46%) patients in the IA group (50%) exhibited allo-immunization after LTx vs. 70 (53%) (p = 0.4), and acute AMR and ACR affected 22 (11%) and 62 patients (30%) in the first year, with no difference between the 2 groups (Supplementary Figure S4). CLAD, mainly BOS (74% of all CLAD diagnoses), was diagnosed in 48 patients (23%) (Supplementary Tables S11-S13). Multi-state modelling did not demonstrate a difference in probability of CLAD occurrence between the 2 groups at 36 months (Supplementary Figure S2).

### FEV1 trajectories

Patients reached their best median FEV1% of 76 (IQR 62–88) % predicted after a median of 177 (87–405) days post-LTx. On time point analysis, FEV1% during the first 3 months was lower for patients with recent IA exposure (Supplementary Figure S5), and linear-mixed modelling of FEV1 during the first year confirmed that IA exposure negatively affected FEV1 evolution over time (effect size: 0.77, 95%CI [0.63–0.96], random effect standard deviation: 0.04). ANOVA showed that including IA exposure status as a fixed effect provided improved the model performance (p = 0.027).

To explain this difference in early evolution in FEV1, we sought to analyse the relation between perioperative infections and respiratory function. The number of pneumonia episodes in the first 6 months was negatively correlated with FEV1 at 6, 12, and 24 months (r = −0.45 to −0.57, p < 0.001 for all) (Supplementary Figure S6).

## Discussion

To date and to our knowledge, this is the first study to demonstrate that patients with ILD who received non-steroidal IA during the year before LTx exhibited increased mortality in the first 12 months after the procedure. These patients had more pneumonia episodes and were at increased risk of grade 3 PGD, bronchovascular fistulas, CMV viremia under prophylaxis and ganciclovir-resistant CMV. For patients with recent IA exposure, pulmonary function was significantly worse during the first months after LTx and remained persistently negatively correlated over time with the number of pneumonia episodes experienced in the first 6 months.

The main findings of our study align with previous research suggesting a detrimental effect of pre-LTx IA on post-LTx outcomes in patients with ILD. In a cohort of CTD-ILD patients undergoing LTx, postoperative infections were more numerous and earlier for those exposed [13]. In another cohort of 286 patients with ILD, those who received corticosteroids or IA without antifibrotics experienced more intraoperative blood loss, longer mechanical ventilation and more frequent grade 3 PGD than those not exposed, but with no difference in mortality [14].

We believe that our selection criteria enabled us to better discriminate the specific effect of pre-LTx non-steroidal IA on outcomes. Although we chose to consider any IA exposure within 12 months of LTx as significant, because the effects of these treatments can persist even after their discontinuation [9, 10, 15], we separately analysed IA without systemic corticosteroids. Hence, we could quantify the yearly cumulative dose of corticosteroids patients received, which was used to adjust multivariate analyses, to better individualize these risk factors. This approach was applied successfully to assess corticosteroid-related adverse events and immunodeficiency in rheumatological diseases [16] and asthma, with most adverse outcomes occurring at cumulative exposures of 1 g per year [17]. In our study, cumulative corticosteroid exposure was associated with increased mortality on univariate but not multivariate analysis, thereby suggesting a more serious relative impact of non-steroidal IA exposure.

The higher rates of high emergency transplantation and bilateral procedures in the IA group reflect the clinical trajectory of patients requiring escalated immunosuppression: acute exacerbation of CTD-ILD frequently necessitates high-dose pulse corticosteroids or rituximab as a bridge, and this same clinical deterioration drives listing prioritisation. Both high emergency status and acute exacerbation were included in the multivariable model; the persistence of the independent IA effect after their adjustment, and its confirmation in the IPTW analysis indicate that the association exceeds what can be attributed to measurable pre-LTx severity alone.

The two most common causes of early mortality after LTx are PGD and infection [1], two complications more frequently observed in patients with recent IA exposure in our study. PGD is a form of acute lung injury after LTx characterized by hypoxemia and infiltrates seen on chest radiography occurring within 72 h^12^. Known recipient- and procedure-specific risk factors include obesity, PH, operating time, and surgical procedure complexity [18]. In our study, patients exposed to IA also had higher rates of intraoperative VA-ECMO and more red blood cell transfusions than those not exposed, which suggests more difficult procedures.

Several mechanisms may account for the higher rates of grade 3 PGD and bronchovascular fistula in IA-exposed patients. Regarding PGD: cytotoxic agents deplete myeloid progenitors and may impair the innate immune response required for alveolar repair after ischemia-reperfusion injury, even after clinical discontinuation. Systemic corticosteroids, at significantly higher cumulative doses in the IA group, cause protein catabolism, sarcopenia, and impaired tissue repair capacity through suppression of fibroblast activity and angiogenesis, compromising both post-reperfusion recovery and anastomotic wound healing [19–21]. Regarding bronchovascular fistula: anastomotic healing depends on fibroblast proliferation, collagen synthesis, and bronchial arterial neovascularisation, all of which are inhibited by corticosteroids and cytotoxic agents [21]. An indirect pathway is equally plausible: severe PGD compromises bronchial arterial reconstitution, creating ischaemic anastomotic conditions, while the higher burden of peri-operative infections in the IA group may further weaken anastomotic integrity.

Longitudinal analysis showed that patients with IA exposure had significantly worse pulmonary function in the first months after LTx than those not exposed and that FEV1 remained negatively correlated with the number of pneumonia episodes during the first 6 months at 6, 12, and 24 months. Considering the prognostic value of early FEV1 values after LTx [22], these patients may exhibit increased respiratory vulnerability at a critical phase of their clinical course.

The overall breakthrough viremia rate of 13% in the CMV-at-risk population is consistent with recently published rates in the CTOT-20 prospective cohort [23]. The higher rate in the IA group (21% vs. 5.2%) likely reflects entry into transplantation with impaired CMV-specific T-cell immunity following immunosuppressive therapy. The enrichment of ganciclovir-resistant CMV in the IA group (14% of CMV-at-risk IA-exposed patients) is mechanistically consistent: impaired T-cell control permits higher viral burdens at breakthrough, increasing selective pressure for UL97 kinase mutations; prolonged prophylaxis duration in high-risk patients further compounds this [24, 25].

Despite the higher intraoperative transfusion burden in the IA group, a recognised driver of HLA sensitization, preformed DSA prevalence and allo-immunization rates were comparable between groups, numerically lower in the IA group, potentially consistent with the B-cell suppressive effects of rituximab and cytotoxic agents. These findings argue against differential HLA sensitisation as a primary explanation for the observed outcome differences.

Finally, we found no significant association between IA exposure and allo-immunization, ACR, AHR or CLAD occurrence, probably because these treatments do not increase immunoreactivity and had no link to risk factors for these complications.

One limitation of our study is its monocentric and retrospective design, although this allowed us to compare a more homogeneous population in terms of follow-up and post-transplant management in a field in which therapeutic approaches are highly centre-dependent. Overall survival was lower than in some other cohorts and international registry data [1, 26, 27], but was in line with the most recent statement from the French *Agence de Biomédecine* (2023 report) which stated a similar nation-wide 1-year survival rate of 71% and a median survival of 55 months for patients undergoing LTx for pulmonary fibrosis. The slightly lower median survival in our cohort may be linked to the high rate of high-emergency and single LTx procedures, reflecting more severe ILD, challenging conditions for LTx, and a high proportion of older patients with comorbidities–however, these factors were used in adjusting multivariate analysis to individualize IA-related risk. The Lung Allocation Score was not available as an adjusting covariate, as this cohort was managed within the French allocation system; a standardised international severity comparator is therefore lacking.

The higher prevalence of CTD-ILD in the IA group is a direct reflection of clinical practice: IAs are the established disease-modifying therapy for most CTD-ILD subtypes, and their use is therefore intrinsic to the management of this diagnosis. Importantly, CTD-ILD was not an independent predictor of survival in the univariate Cox model (HR 1.40, 95% CI 0.64–3.06, p = 0.40 vs. IPF), and ILD subtype was not retained in the multivariable model, a decision which was supported by the results of the IPTW analysis. Residual confounding from CTD-specific comorbidities not captured in the dataset, such as cardiac involvement in systemic sclerosis or oesophageal dysmotility, cannot be excluded. Finally, the IA group was exposed to agents with markedly different mechanisms of action, pharmacokinetic profiles, and durations of immunosuppressive effect; the pooled analysis captures the clinical phenotype of recent IA exposure but precludes drug class–specific conclusions. The duration of individual exposures and the potential additive immunosuppressive effects of combination regimens (present in 22% of IA-exposed patients) could also not be formally quantified.

Overall, our results support considering IA exposure during the selection of LTx candidates among patients with ILD, to limit exposure whenever possible. Raising the awareness of clinicians and researchers to this risk factor may help inform management decisions at multiple levels: 1) in the context of worsening disease, the decision to escalate to IA should be carefully considered, especially for diseases for which these drugs have weaker evidence (e.g., idiopathic interstitial pneumonias) [28, 29]; 2) during the pre-LTx evaluation process, considering IA exposure may help stratify risk in candidates; and 3) among patients with an active LTx project, reducing or stopping IA may be discussed, particularly if the patient could benefit or already benefits from an antifibrotic agent in that antifibrotic therapies have now demonstrated their efficacy on the disease course and exacerbations in progressive pulmonary fibrosis beyond IPF [30, 31] and show no evidence of increasing post-LTX adverse outcomes [6, 7]. However, the use of IA in an eligible patient should not be wholly restricted because responders may find their LTx project delayed or even cancelled.

## Conclusion

IA exposure in the year preceding LTx was an independent risk factor for 12-month mortality among patients with ILD and predisposed them to early complications, including severe PGD, bronchovascular fistulas, pneumonia and the emergence of ganciclovir-resistant CMV. Considering IA exposure during pre-LTx assessment process may improve risk stratification in these patients and guide therapeutic decisions. Further prospective and multicentric studies are needed to confirm these findings.

## Data Availability

The original contributions presented in the study are included in the article/Supplementary Material, further inquiries can be directed to the corresponding author.

## Footnotes

1
http://www.r-project.org/
