# Supplementary material for: Impact of pre-transplantation exposure to immunosuppressive agents on lung transplant outcomes in interstitial lung disease
Source: Transpl Int. 2026 Jun 10;39:16549. doi: 10.3389/ti.2026.16549 (PMC13321801; doi:10.3389/ti.2026.16549)
Supplement: Supplementary file 1 [file DataSheet1.docx]

**Supplementary Materials**

**Supplementary Methods**

**Lung transplantation (LTx) procedure at Bichat Lung Transplantation Center.** Patients received a bolus of corticosteroids in the operating room, and post-LTx immunosuppressive agents (IAs) were initiated shortly after surgery. Before May 2024, LTx was performed without induction therapy; afterwards, either Basiliximab or anti-thymocyte globulin were used depending on pre-LTx alloimmunization. The maintenance regimen typically included tacrolimus, myocophenolate mofetil, and systemic corticosteroids started at 0.5 mg/kg per day prednisone equivalent, with gradual tapering (daily dosage decreased by 5 mg every week in the first month until a threshold of 0.25 mg/kg per day, held until the third month, followed by tapering to 0.15 mg/kg per day held until the sixth month, followed by tapering to 7.5 mg per day, continued indefinitely). Anti-human leukocyte antigen levels were measured on the day of LTx and monitored at regular and progressively wider intervals during follow-up. Bronchoscopic surveillance was systematically performed on day 1, day 3, day 7 and once a week for the next month, in addition to for cause procedures. Transbronchial biopsies were taken only in cases of suspected acute rejection. Patients systematically received antifungal prophylaxis with nebulized liposomal amphotericin B and/or an azole antifungal. Cytomegalovirus (CMV) prophylaxis initially involved intravenous ganciclovir followed by oral valganciclovir, for 6 months in recipient-positive (R+) patients and 12 months in case of donor-positive recipient-negative (D+R-) mismatch. For *Pseudomonas aeruginosa* primary infection or recurrent infections with Gram-negative bacilli, patients could receive prolonged antibiotic aerosols.

**High emergency LTx** refers to a prioritization process for patients in imminent danger of death due to end-stage respiratory failure for fibrosing ILD (f-ILD), pulmonary hypertension (PH) and cystic fibrosis. In France, in the context of f-ILD, this procedure is initiated when no obvious contraindications are present. This prioritization ensures rapid access to available donor lungs, which are critically needed, notably in cases of acute exacerbation or progressive worsening leading to severe and persistent hypoxemia despite high-flow oxygen therapy and may require mechanical ventilation or extracorporeal membrane oxygenation to sustain life.

**Supplementary Tables**

**Supplemental Table 1. Immunosuppressive agents (IAs) used during the 12 months before lung transplantation (LTx) (n=76 patients)**

| Number of different IAs used in the 12 months before LTx | |  |
| --- | --- | --- |
|  | 1 | 59 (78) |
|  | 2 | 14 (18) |
|  | 3 | 2 (2.6) |
|  | 4 | 1 (1.3) |
| Non-biologic IA | | 66 (87) |
|  | Cyclophosphamide | 25 (33) |
|  | MMF | 33 (43) |
|  | AZA | 15 (20) |
|  | Tacrolimus | 2 (2.6) |
|  | Tofacitinib | 2 (2.6) |
| Non-biologic IA only* | | 58 (76) |
|  | Cyclophosphamide | 15 (20) |
|  | MMF | 25 (33) |
|  | AZA | 9 (12) |
|  | Tacrolimus | 1 (1.3) |
|  | Tofacitinib | 0 (0) |
| Biologic IA | | 18 (24) |
|  | Rituximab | 15 (20) |
|  | Tocilizumab | 3 (3.9) |
|  | Abatacept | 1 (1.3) |
|  | Anti-TNF | 1 (1.3) |
| Biologic IA only** | | 10 (13) |
|  | Rituximab | 6 (7.9) |
|  | Tocilizumab | 2 (2.6) |
|  | Abatacept | 1 (1.3) |
|  | Anti-TNF | 0 (0) |
| Data are n (%).  IA: immunosuppressive agent; MMF: mycophenolate mofetil; AZA: azathioprine; TNF: tumor necrosis factor  *: includes patients with only non-biologic IA in the 12 months before LTx  **: includes patients with only biologic IA in the 12 months before LTx | | |

**Supplemental Table 2. Lung transplantation-free survival rates at 12, 24 and 36 months for patients overall and those exposed or not to immunosuppressive agents (IAs) < 12 months before LTx (n = 209)**

| Characteristics | 12 months | 24 months | 36 months |
| --- | --- | --- | --- |
| Overall | 74% (68–80) | 60% (54–68) | 54% (47–61) |
| IA exposure < 12 months | 80% (74–87) | 65% (57–74) | 56% (48–66) |
| No IA exposure < 12 months | 62% (52–74) | 53% (43–66) | 49% (39–63) |

Data are percentage (95% confidence interval).

**Supplemental Table 3. Covariate balance before and after inverse probability of treatment weighting (IPTW).**

| Covariate | SMD before | SMD after | Balanced |
| --- | --- | --- | --- |
| ILD subtype: IPF | -0.449 | -0.081 | Yes |
| ILD subtype: CTD-ILD | 0.365 | 0.072 | Yes |
| ILD subtype: fHP | 0.027 | 0.037 | Yes |
| ILD subtype: fNSIP | 0.074 | 0.006 | Yes |
| ILD subtype: uILD | 0.056 | -0.016 | Yes |
| ILD subtype: Other | -0.073 | -0.019 | Yes |
| Severe pulmonary hypertension | 0.042 | 0.065 | Yes |
| Acute ILD exacerbation (<12 mo) | 0.106 | 0.044 | Yes |
| High emergency transplantation | 0.153 | 0.049 | Yes |
| Annual corticosteroid dose (g) | 0.891 | 0.34 | No |
| Age at transplantation (years) | -0.384 | -0.146 | No |
| BMI (kg/m2) | 0.342 | 0.023 | Yes |
| SMD: standardised mean difference. Values <0.10 indicate adequate balance. Corticosteroid dose and age remained imperfectly balanced after weighting (SMD 0.34 and 0.15, respectively); corticosteroid dose is near-collinear with IA exposure by design. IA: immunosuppressive agent; ILD: interstitial lung disease; fHP: fibrotic hypersensitivity pneumonia; CTD-ILD: connective tissue disease-associated ILD; fNSIP: fibrotic non-specific interstitial pneumonia; uILD: unclassified ILD; BMI: body mass index. | | | |

**Supplemental Table 4. Pairwise comparison of immunosuppressive agent (IA) types**

| Comparison | HR | 95% CI | p-value |
| --- | --- | --- | --- |
| Biologic IA* vs. none | 3.01 | 1.09–8.33 | 0.033 |
| Non-biologic IA ** vs. none | 1.97 | 1.05–3.67 | 0.034 |
| Non-biologic IA vs. biologic IA | 1.53 | 0.57–4.14 | 0.398 |
| HR: hazard ratio; CI: confidence interval; LTx: lung transplantation; PH: pulmonary hypertension  *: rituximab, tocilizumab or abatacept without non-biological agent in the 12 months before LTx  **: mycophenolate mofetil, azathioprine, cyclophosphamide, methotrexate, tofacitinib or tacrolimus without biological agent in the 12 months before LTx  Adjusted for confounders: IA type, PH, corticosteroid cumulative dose (last 12 months) (g) | | | |

**Supplemental Table 5. Multistate model analysis of chronic lung allograft dysfunction (CLAD)-free survival**

| Event | Time | Probability (%)  (recent IA) | Probability (%)  (no recent IA) | Difference  (95% CI) | p-value |
| --- | --- | --- | --- | --- | --- |
| Death | 3 years | 48 | 35.5 | 12.4 (0.9, 23.9) | 0.034 |
| CLAD | 3 years | 15 | 16.1 | -1.1 (-10.3, 8) | 0.8 |
| IA: immunosuppressive agent; CI: confidence interval | | | | | |

**Supplemental Table 6. Univariate analysis of odds of grade 3 primary graft dysfunction***

|  | | Univariate analysis | | | | | | |
| --- | --- | --- | --- | --- | --- | --- | --- | --- |
| Characteristics | | N | | **OR** | | **95% CI** | | **p-value** |
| IA exposure < 12 months | | 209 | | 3.20 | | 1.42–7.45 | | **0.005** |
| Corticosteroids exposure | |  | |  | |  | |  |
|  | Cumulative dose (last 12 months) (g) | 200 | 1.10 | | 0.97–1.24 | | 0.13 | |
|  | At transplantation (mg) | 209 | 1.00 | | 0.98–1.02 | | 0.9 | |
| Antifibrotics at transplantation | | 209 | | 0.67 | | 0.28–1.52 | | 0.4 |
| Male sex | | 209 | | 0.76 | | 0.32–1.93 | | 0.5 |
| Age at transplantation (years) | | 209 | | 0.96 | | 0.92–1.00 | | **0.038** |
| BMI ≥ 30 (kg/m^2^) | | 209 | | 2.08 | | 0.76–5.22 | | 0.13 |
| Severe pulmonary hypertension | | 204 | | 1.15 | | 0.17–4.57 | | 0.9 |
| ILD etiology | | 209 | |  | |  | |  |
|  | IPF |  | — | | — | |  | |
|  | CTD-ILD |  | 1.76 | | 0.49–5.94 | | 0.4 | |
|  | f-HP |  | 1.67 | | 0.41–5.99 | | 0.4 | |
|  | f-NSIP |  | 1.88 | | 0.38–7.51 | | 0.4 | |
|  | uILD |  | 11.3 | | 3.29–41.2 | | **<0.001** | |
|  | Other |  | 0.66 | | 0.03–4.09 | | 0.7 | |
| ILD exacerbation < 12 months | | 209 | | 1.51 | | 0.66–3.39 | | 0.3 |
| Type of LTx | | 209 | |  | |  | |  |
|  | Single |  | — | | — | |  | |
|  | Double |  | 4.55 | | 1.78–14.0 | | **0.003** | |
| High emergency transplantation | | 209 | | 1.09 | | 0.44–2.49 | | 0.9 |
| Number of RBC pack transfusions | | 207 | | 1.19 | | 1.07–1.35 | | **0.003** |
| Donor age (years) | | 200 | | 1.01 | | 0.99–1.04 | | 0.3 |
| IA: immunosuppressive agent; OR: odds ratio; CI: confidence interval; BMI: body mass index; ILD: interstitial lung disease; IPF: idiopathic pulmonary fibrosis; f-HP: fibrotic hypersensitivity pneumonia; CTD-ILD: connective tissue disease associated interstitial lung disease; f-NSIP: fibrotic non-specific interstitial pneumonia; uILD: unclassified interstitial lung disease; LTx: lung transplantation; RBC: red blood cell  *: We defined grade 3 primary graft dysfunction as a persistent PaO2/FiO2 ratio < 200 or extracorporeal membrane oxygenation requirement within 48 to 72 hr after LTx or death occurring within the first 48 hr with grade 3 primary graft dysfunction. | | | | | | | | |

**Supplemental Table 7. Univariate analysis of odds of bronchovascular fistula (n=6 events)**

|  | | Univariate analysis | | | | | | |
| --- | --- | --- | --- | --- | --- | --- | --- | --- |
| Characteristics | | N | | **OR** | | **95% CI** | | **p-value** |
| IA exposure < 12 months | | 209 | | 9.43 | | 1.48–183 | | **0.042** |
| Corticosteroids exposure | |  | |  | |  | |  |
|  | Cumulative dose (last 12 months) (g) | 200 | 0.98 | | 0.70–1.24 | | 0.9 | |
|  | At transplantation (mg) | 209 | 1.51 | | 0.29–11.1 | | 0.6 | |
| Antifibrotics at transplantation | | 209 | | 0.73 | | 0.10–3.84 | | 0.7 |
| Male sex | | 209 | | 0.62 | | 0.12–4.60 | | 0.6 |
| Age at transplantation (years) | | 209 | | 0.98 | | 0.91–1.07 | | 0.6 |
| BMI ≥ 30 (kg/m^2^) | | 209 | | 1.10 | | 0.06–7.15 | | >0.9 |
| Severe pulmonary hypertension* | | 209 | | 0.00 | |  | |  |
| ILD etiology | | 209 | |  | |  | |  |
|  | IPF |  | — | | — | |  | |
|  | CTD-ILD |  | 2.40 | | 0.28–20.7 | | 0.4 | |
|  | f-HP |  | 2.90 | | 0.34–25.0 | | 0.3 | |
|  | f-NSIP***** |  | 0.00 | |  | |  | |
|  | uILD***** |  | 0.00 | |  | |  | |
|  | Other***** |  | 0.00 | |  | |  | |
| ILD exacerbation < 12 months | | 209 | | 1.97 | | 0.36–10.9 | | 0.4 |
| Type of transplantation | | 209 | |  | |  | |  |
|  | Single LTx |  | — | | — | |  | |
|  | Double LTx |  | 0.82 | | 0.15–4.52 | | 0.8 | |
| High emergency transplantation | | 209 | | 0.45 | | 0.02–2.88 | | 0.5 |
| PGD grade | | 209 | | 1.49 | | 0.76–2.62 | | 0.2 |
| Postoperative VA-ECMO | | 209 | | 0.76 | | 0.04–4.89 | | 0.8 |
| IA: immunosuppressive agent; OR: odds ratio; CI: confidence interval; BMI: body mass index; ILD: interstitial lung diseases; IPF: idiopathic pulmonary fibrosis; f-HP: fibrotic hypersensitivity pneumonia; CTD-ILD: connective tissue disease associated interstitial lung disease; f-NSIP: fibrotic non-specific interstitial pneumonia; uILD: unclassified interstitial lung disease; LTx: lung transplantation; PGD: primary graft dysfunction; VA: veno-arterial; ECMO: extracorporeal membrane oxygenation.  *Impact of severe pulmonary hypertension, f-NSIP, uILD could not be assessed as none of the patients with bronchovascular fistulas exhibited these conditions. | | | | | | | | |

**Supplemental Table 8. Infectious outcomes (non-cytomegalovirus) in the first 6 months after lung transplantation**

|  | | Total | Recent IA | No recent IA | p-value | |  |
| --- | --- | --- | --- | --- | --- | --- | --- |
| Characteristics | | 156 | 47 | 109 |  | |  |
| Infectious outcomes in the first 6 months | |  |  |  |  | | |
|  | Number of pneumonia infections | 2.00 (1.00-3.00) | 2.00 (1.00-4.00) | 1.00 (0.50-2.50) | **0.001** |  |  |
|  | Number of antibiotics courses | 3.00 (2.00-5.00) | 4.00 (3.00-5.00) | 3.00 (1.00-5.00) | **0.010** |  |  |
|  | Invasive fungal infection | 13 (8.4) | 6 (13) | 7 (6.5) | 0.2 |  |  |
| Other infectious outcomes | |  |  |  |  | | |
|  | *Pseudomonas aeruginosa* infection | 106 (68) | 34 (72) | 72 (66) | 0.4 |  |  |
|  | *Clostridium* infection | 11 (7.1) | 3 (6.4) | 8 (7.3) | >0.9 |  |  |
| Data are n (%) or median (interquartile range).  IA: immunosuppressive agent | | | | | | |  |

**Supplemental Table 9. Cumulative incidence of cytomegalovirus reactivation under prophylaxis for recipient-positive and donor-positive recipient-negative patients during the first 12 months after lung transplantation by immunosuppressive agent (IA) exposure (n = 152)**

| Characteristics | | Month 6 | Month 12 | p-value |
| --- | --- | --- | --- | --- |
| IA exposure < 12 months | |  |  | **0.005** |
|  | False | 1.2% (0.10–5.9) | 5.2% (1.7–12) |  |
|  | True | 18% (8.3–31) | 21% (10–34) |  |
| Data are percentage (95% confidence interval).  IA: immunosuppressive agent | | | | |

**Supplemental Table 10. Cytomegalovirus complications by immunosuppressive agent (IA) exposure**

|  | | Total | Recent IA | No recent IA | p-value |
| --- | --- | --- | --- | --- | --- |
| Pooled R+ and D+/R- patients | | n=152 | n=58 | n=94 |  |
|  | CMV reactivation after prophylaxis | 60 (40) | 21 (37) | 39 (41) | 0.6 |
|  | CMV reactivation under prophylaxis | 20 (13) | 12 (21) | 8 (8.5) | **0.028** |
|  | Ganciclovir-resistant CMV | 11 (7.3) | 8 (14) | 3 (3.2) | **0.021** |
| R+ patients | | n=128 | n=49 | n=79 |  |
|  | CMV reactivation after prophylaxis | 49 (39) | 17 (35) | 32 (41) | 0.7 |
|  | CMV reactivation under prophylaxis | 9 (7.1) | 6 (13) | 3 (3.8) | 0.081 |
|  | Ganciclovir-resistant CMV | 3 (2.4) | 3 (6.3) | 0 (0) | 0.052 |
| D+R- patients | | n=24 | n=9 | n=15 |  |
|  | CMV reactivation after prophylaxis | 11 (46) | 4 (44) | 7 (47) | >0.9 |
|  | CMV reactivation under prophylaxis | 11 (46) | 6 (67) | 5 (33) | 0.2 |
|  | Ganciclovir-resistant CMV | 8 (33) | 5 (56) | 3 (20) | 0.10 |
| Data are n (%).  CMV: cytomegalovirus; R+: positive recipient; R-; negative recipient; D+: positive donor | | | | | |

**Supplemental Table 11. Immunological outcomes and chronic lung allograft dysfunction (CLAD) by immunosuppressive agent (IA) exposure**

|  | | Total | Recent IA | No recent IA | p-value |
| --- | --- | --- | --- | --- | --- |
| Characteristics | | 209 | 76 | 133 |  |
| Acute rejection | |  |  |  |  |
|  | Pre-transplantation immunization | 48 (24) | 14 (19) | 34 (27) | 0.2 |
|  | Alloimmunization | 105 (50) | 35 (46) | 70 (53) | 0.4 |
|  | ACR < 12 months | 62 (30) | 18 (24) | 44 (33) | 0.14 |
|  | Acute AMR < 12 months | 22 (11) | 11 (15) | 11 (8.3) | 0.2 |
| CLAD | | 48 (23) | 16 (21) | 32 (24) | 0.6 |
| CLAD type | |  |  |  | 0.8 |
|  | BOS | 35 (74) | 10 (67) | 25 (78) |  |
|  | Unclassifiable | 3 (6.4) | 1 (6.7) | 2 (6.3) |  |
|  | Mixed | 3 (6.4) | 1 (6.7) | 2 (6.3) |  |
|  | RAS | 6 (13) | 3 (20) | 3 (9.4) |  |
| Data are n (%).  ACR: acute cellular rejection; AMR: antibody-mediated rejection; BOS: bronchiolitis obliterans syndrome; RAS; restrictive allograft syndrome | | | | | |

**Supplemental Table 12. Cumulative incidence of acute cellular rejection during the first 12 months after lung transplantation by immunosuppressive agent (IA) exposure (n = 208)**

|  | | Month 6 | Month 12 | p-value |
| --- | --- | --- | --- | --- |
| IA exposure < 12 months | |  |  | 0.2 |
|  | False | 30% (23–38) | 33% (25–41) |  |
|  | True | 22% (14–32) | 24% (15–34) |  |
| Data are percentage (95% confidence interval). | | | | |

**Supplemental Table 13. Cumulative incidence of acute antibody-mediated rejection during the first 12 months after lung transplantation by immunosuppressive agent (IA) exposure (n = 208)**

|  | | Month 6 | Month 12 | p-value |
| --- | --- | --- | --- | --- |
| IA exposure < 12 months | |  |  | 0.2 |
|  | False | 8.3% (4.4–14) | 8.3% (4.4–14) |  |
|  | True | 15% (7.8–24) | 15% (7.8–24) |  |

Data are percentage (95% confidence interval).

**Supplementary Figures**

**
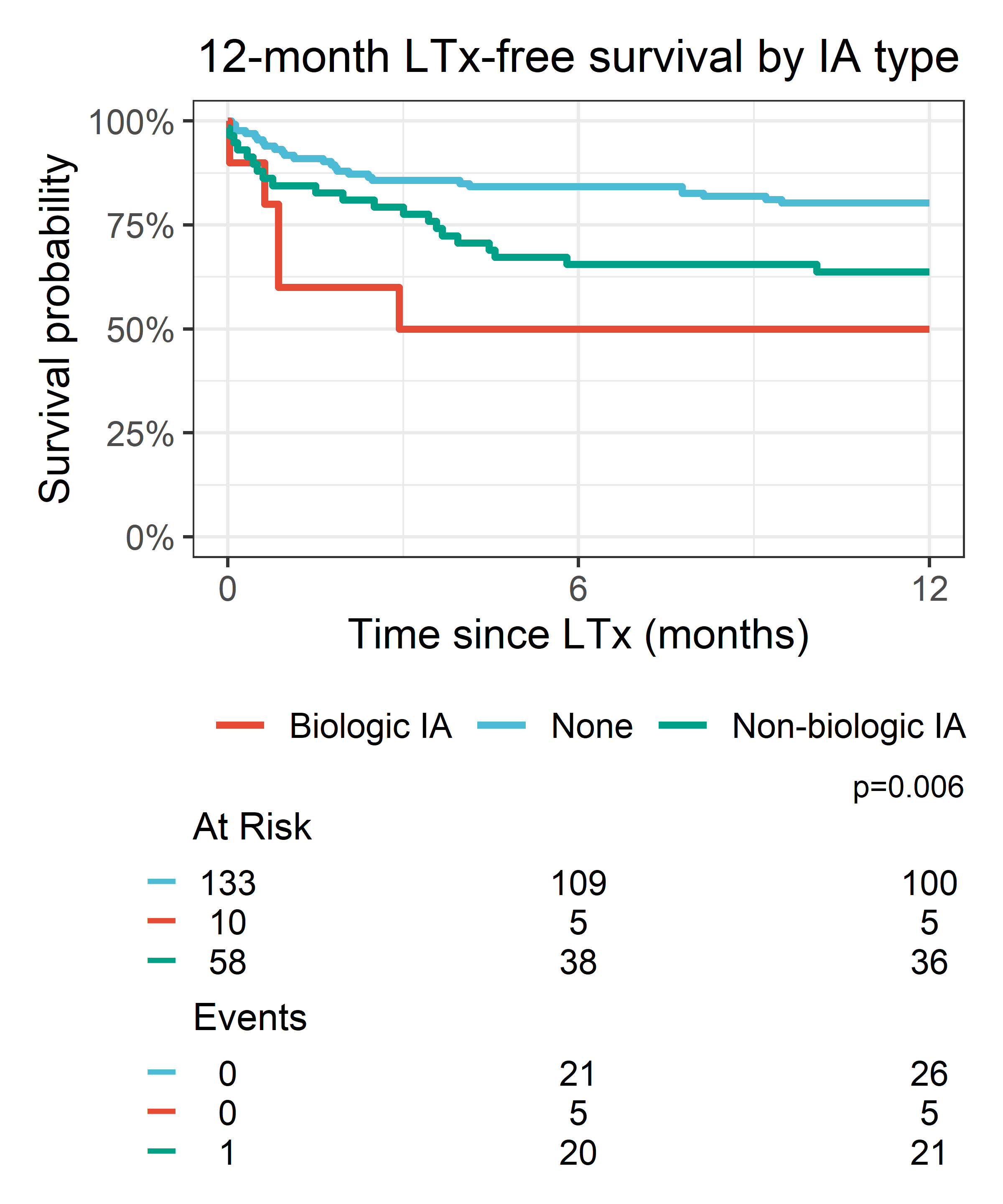
**

**Supplemental Figure 1. Twelve-month lung transplantation (LTx)-free survival by immunosuppressive agent (IA) type (Kaplan-Meier curves).**

**
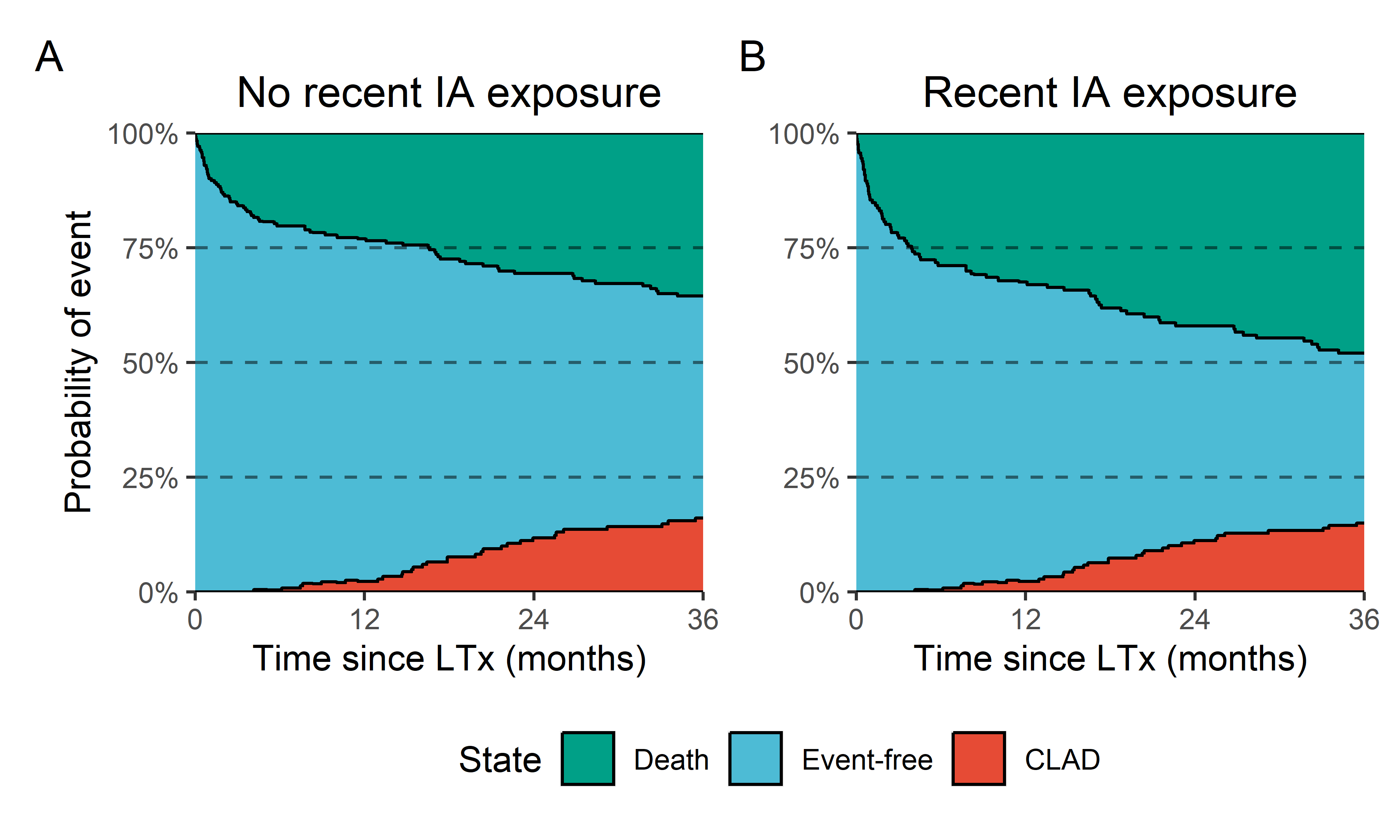
**

**Supplemental Figure 2. Graphical representation of transition probability to chronic lung allograft dysfunction (CLAD) or death over time (multi-state model).** LTx: lung transplantation; IA: immunosuppressive agent

**
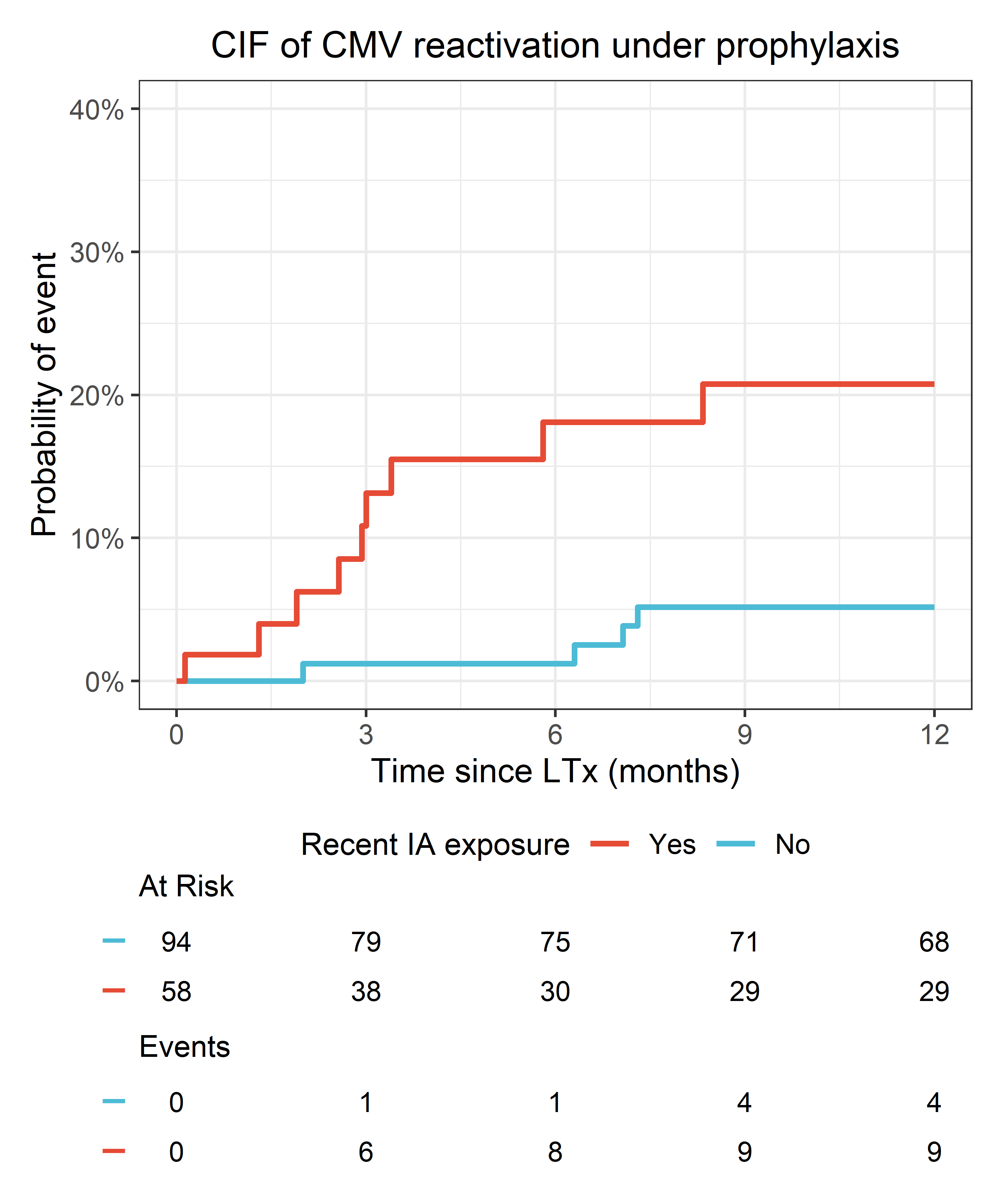
**

**Supplemental Figure 3. Cumulative incidence function of cytomegalovirus (CMV) reactivation under prophylaxis in at-risk patients (n=152) by immunosuppressive agent (IA) exposure status.** LTx: lung transplantation; recent exposure: < 12 months before LTx; CIF: cumulative incidence function

**
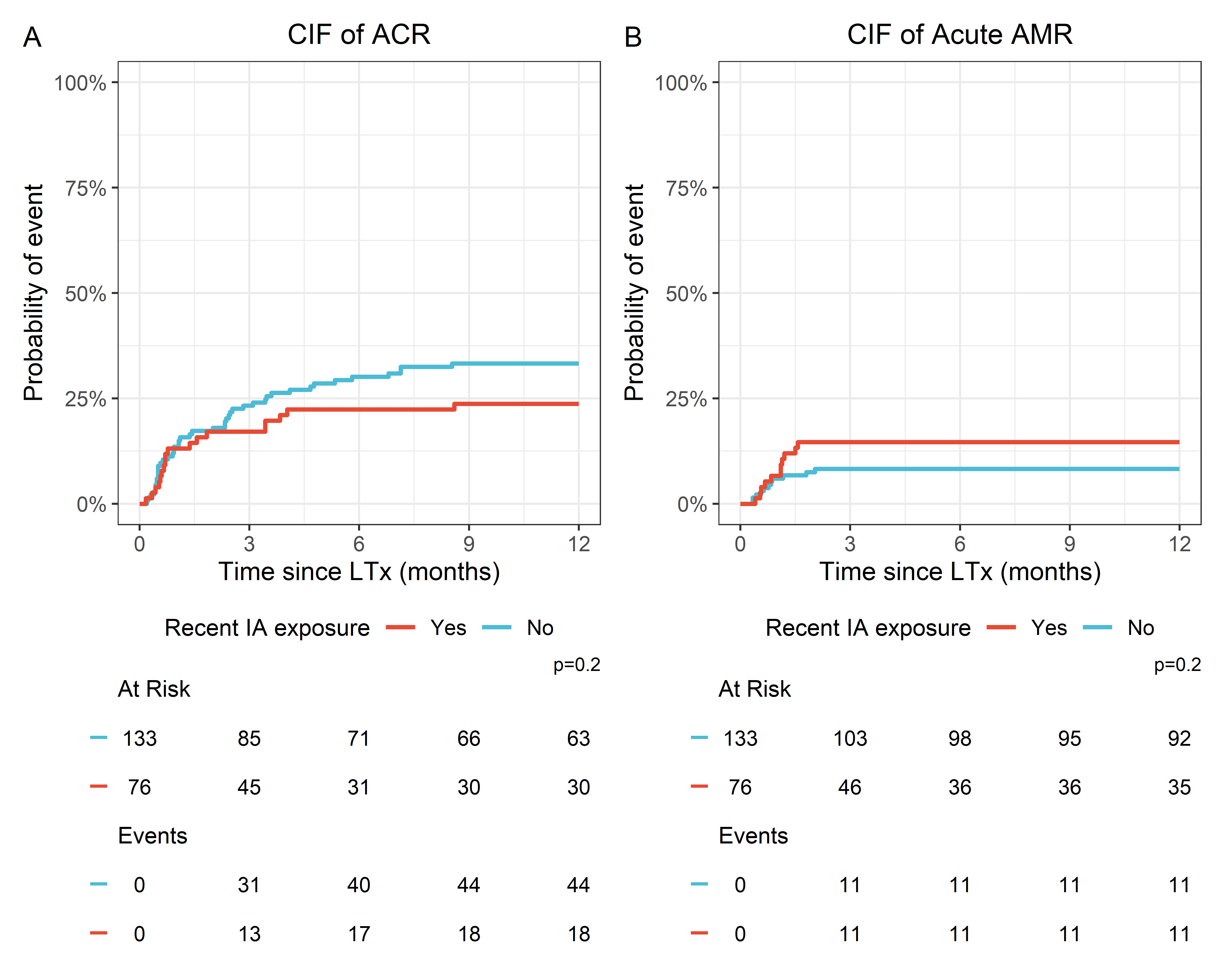
**

**Supplemental Figure 4. Cumulative incidence of acute cellular rejection (ACR) and acute antibody-mediated rejection (AMR) in the first year after lung transplantation (LTx).**

**
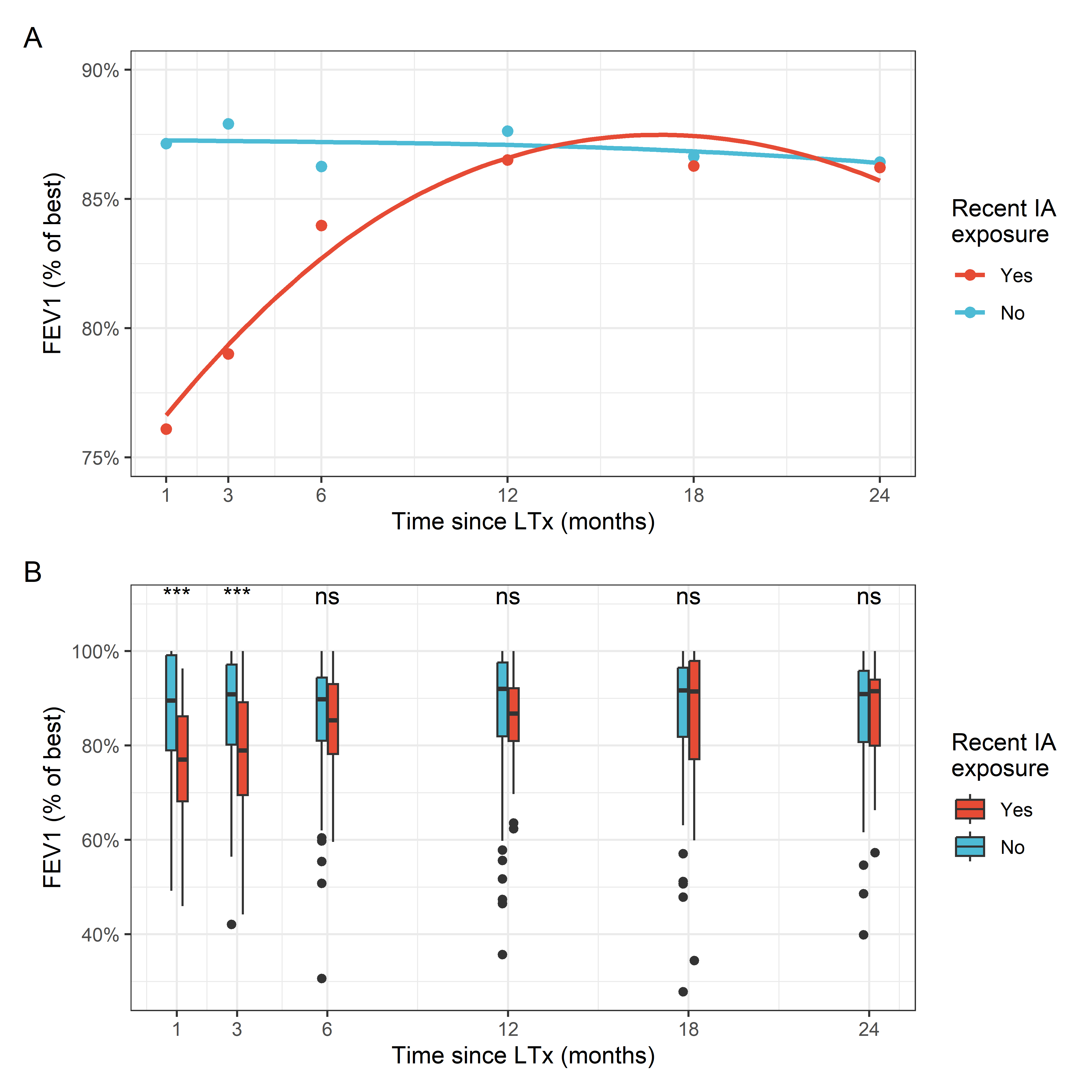
**

**Supplemental Figure 5. Forced expiratory volume in 1 s (FEV1) evolution (as a percentage of best post-lung transplantation [LTx] value) during the first 24 months after LTx, according to immunosuppressive agent (IA) exposure status.** A: FEV1 trajectories in study population using mean values at each time point (points) and polynomial regression (curves). B: Box plots of FEV1 values at each time point. Recent exposure: < 12 months before LTx; ns: not significant. ***P<0.001

**
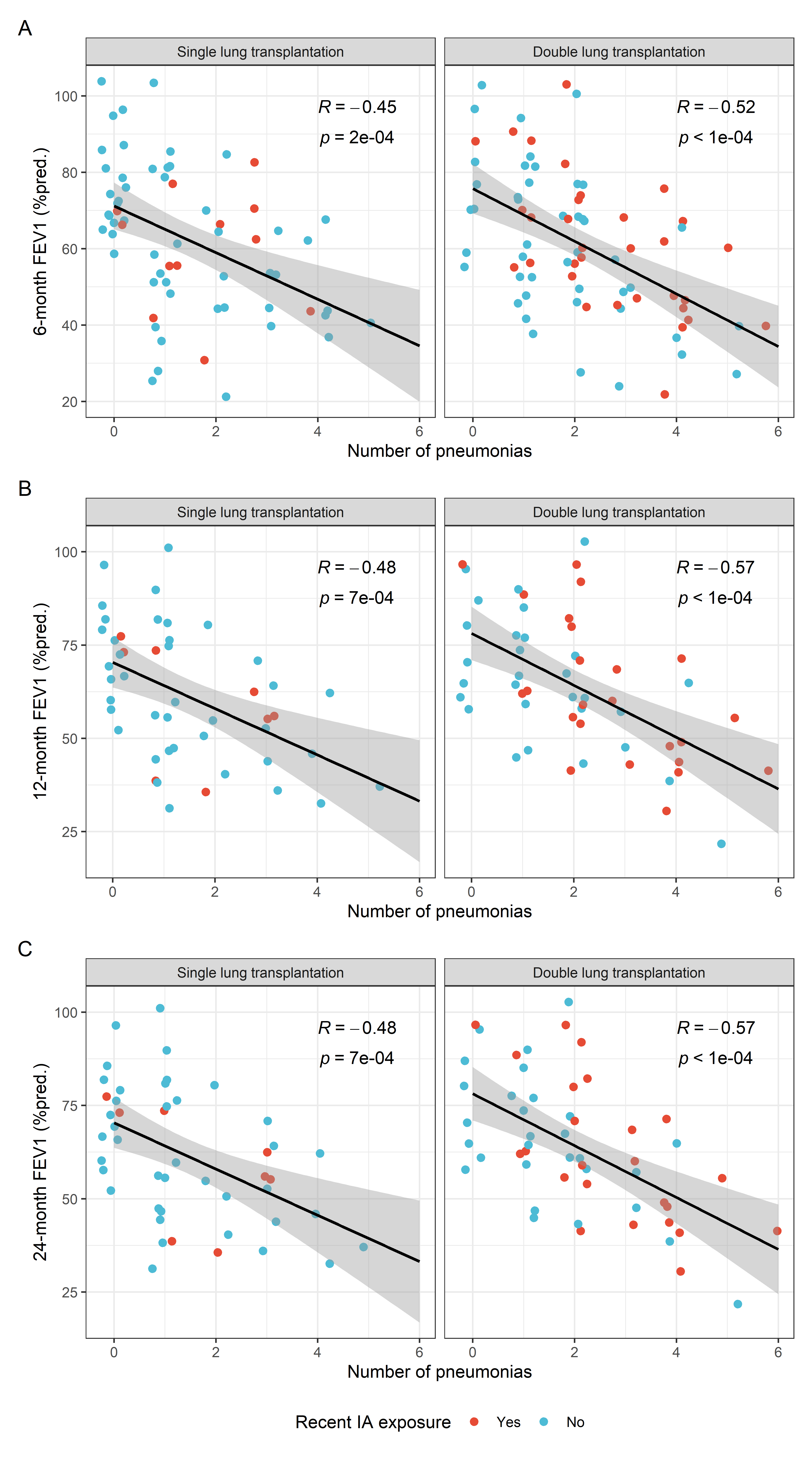
**

**Supplemental Figure 6. Correlations between number of pneumonia infections and forced expiratory volume in 1 s (FEV1; %predicted) at A) 6, B) 12 and C) 24 months after lung transplantation (LTx).** Sample size for double LTx: n = 108, 113 and 103 (A, B and C, respectively); single LTx: n = 86, 93 and 91 (A, B and C respectively). IA: immunosuppressive agent
